# *In vivo* 3D analysis of systemic effects after local heavy-ion beam irradiation in an animal model

**DOI:** 10.1038/srep28691

**Published:** 2016-06-27

**Authors:** Kento Nagata, Chika Hashimoto, Tomomi Watanabe-Asaka, Kazusa Itoh, Takako Yasuda, Kousaku Ohta, Hisako Oonishi, Kento Igarashi, Michiyo Suzuki, Tomoo Funayama, Yasuhiko Kobayashi, Toshiyuki Nishimaki, Takafumi Katsumura, Hiroki Oota, Motoyuki Ogawa, Atsunori Oga, Kenzo Ikemoto, Hiroshi Itoh, Natsumaro Kutsuna, Shoji Oda, Hiroshi Mitani

**Affiliations:** 1 Department of Integrated Biosciences, Graduate School of Frontier Sciences, The University of Tokyo, Chiba, Japan; 2 Takasaki Advanced Radiation Research Institute, Quantum Beam Science Research Directorate, National Institutes for Quantum and Radiological Science and Technology, Gunma, Japan; 3 Department of Anatomy, Kitasato University School of Medicine, Kanagawa, Japan; 4 Department of Molecular Pathology, Yamaguchi University Graduate School of Medicine, Yamaguchi, Japan; 5 LPixel Inc., Tokyo, Japan

## Abstract

Radiotherapy is widely used in cancer treatment. In addition to inducing effects in the irradiated area, irradiation may induce effects on tissues close to and distant from the irradiated area. Japanese medaka, *Oryzias latipes*, is a small teleost fish and a model organism for evaluating the environmental effects of radiation. In this study, we applied low-energy carbon-ion (26.7 MeV/u) irradiation to adult medaka to a depth of approximately 2.2 mm from the body surface using an irradiation system at the National Institutes for Quantum and Radiological Science and Technology. We histologically evaluated the systemic alterations induced by irradiation using serial sections of the whole body, and conducted a heart rate analysis. Tissues from the irradiated side showed signs of serious injury that corresponded with the radiation dose. A 3D reconstruction analysis of the kidney sections showed reductions in the kidney volume and blood cell mass along the irradiated area, reflecting the precise localization of the injuries caused by carbon-beam irradiation. Capillary aneurysms were observed in the gill in both ventrally and dorsally irradiated fish, suggesting systemic irradiation effects. The present study provides an *in vivo* model for further investigation of the effects of irradiation beyond the locally irradiated area.

Radiotherapy is widely used in cancer treatment. Exposure to ionizing radiation delivers strong energy to the target and has become an effective cancer therapy in recent years^1^,^2^. Irradiation to a particular area may cause radiation effects on nearby and distant tissues in addition to the irradiated area^3^,^4^. Therefore, it is important to evaluate the effects on both irradiated and other non-irradiated parts of the body. *In vitro* cell culture has been used to study the effects of irradiation in both the irradiated cells and neighbouring cells; the effect on non-irradiated cells is called the “bystander effect”^5^,^6^. However, experiments using cell culture have reported contradictory findings concerning whether a bystander effect and a hormesis effect exist^7^.

Individual recipients of irradiation show multiple classes of effects after irradiation. The non-irradiated cells near irradiated cells or tissues may show effects, and systemic abscopal effects have been reported at multiple locations outside the irradiated volume. The responses of irradiated cells are also mediated by signals from irradiated cells as cohort effects^3^. Several studies have examined radiation effects in specific organs and other responses, including cell death^8^,^9^,^10^,^11^. The risk of irradiation to the cardiovascular system increases after radiotherapy in breast cancer patients^12^. Computerized tomography has long been used to analyse large organs^13^. However, a precise analysis of the cellular responses at the whole-body level has not yet been conducted.

Medaka, *Oryzias latipes*, is a vertebrate animal model with inbred strains that is used to study the acute somatic effects of irradiation at both the cellular and tissue levels^14^,^15^,^16^,^17^,^18^,^19^,^20^. In recent years, medaka has been used to study irradiation in cohort studies^21^. Because of its sensitivity, medaka is also used to examine environmental stresses such as chemicals in addition to radiation^22^,^23^. Several medaka strains with mutations in the chromatophore allow for direct observations of the body’s interior using imaging or videotaping^24^. Medaka is small enough that it can be used to examine the acute somatic effects induced at the cellular level in all tissues. Therefore, it should be relatively simple to observe the status of the organs that are highly sensitive to irradiation in mammals.

Here, we established experimental procedures to locally irradiate adult medaka fish to a depth of approximately 2.2 mm from the body surface by using a heavy-ion beam irradiation system at National Institutes for Quantum and Radiological Science and Technology (QST) in Takasaki. We evaluated the systemic alterations induced by irradiation in serial sections of the whole body, and we performed heart rate analysis after the irradiation. We found evidence of oedema in capillaries in both the irradiated and non-irradiated areas. These observations suggest that the systemic effects are caused by local irradiation.

## Results

### Phenotypes in irradiation-sensitive organs

**Figure 1:**
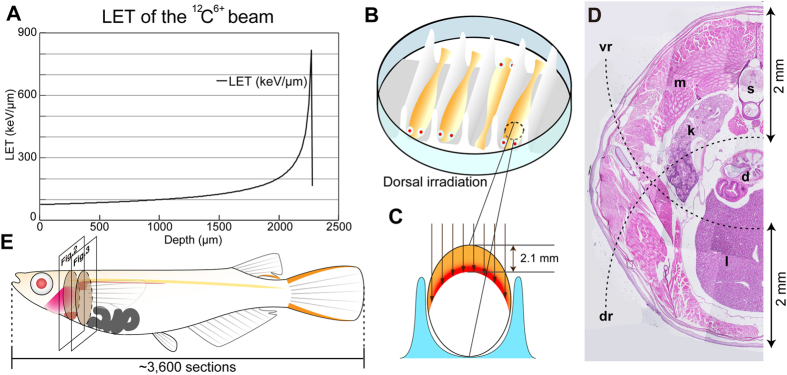
Experimental system for localized irradiation. (**A**) Linear energy transfer for water heavy-ion beam irradiation used in this study. (**B**) Diagram of the positioning of medaka for dorsal irradiation. (**C**) Diagram of the oval-shaped irradiated area. (**D**) HE staining of transverse section around the kidney in adult SK2 medaka. Dashed lines show a distance of approximately 2 mm from the dorsal or ventral surface. (**E**) Diagram of a medaka fish indicating the number of transverse serial sections. d, digestive organ; dr, range of dorsal irradiation; k, kidney; l, liver; LET, linear energy transfer; m, muscle; s, spinal cord; vr, range of ventral irradiation.

A carbon-ion beam accelerated with an Azimuthally Varying Field cyclotron of Takasaki Ion Accelerator for Advanced Radiation Application (TIARA; Takasaki, Japan), QST, was used for heavy-ion beam irradiation of the fish. The range of the carbon particles was approximately 2.2 mm in water (Fig. 1A). Anaesthetized adult fish were aligned in a petri dish with the dorsal (Fig. 1B) or ventral side facing upward for dorsal or ventral irradiation, respectively. The cross-sectional shape of the medaka is roughly oval, and we expected that the medaka would be irradiated as shown in Fig. 1C,D. Therefore, we expected that each medaka fish would receive dorsal irradiation (DR) or ventral irradiation (VR) according to its alignment. An analysis of serial sections enabled us to evaluate changes in overall size and shape as well as local changes in organs. For the whole-body analysis of serial sections, we calculated that approximately 5,000 serial sections at a thickness of 5 μm would need to be cut from the head to the tail. In practice, we obtained approximately 3,600 serial sections from each medaka because of shrinkage in the fixative solution (Fig. 1E).

**Figure 2:**
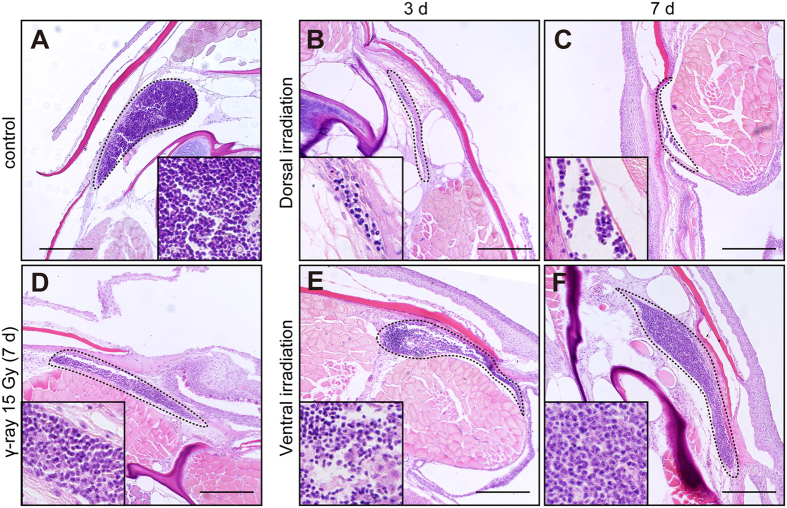
HE staining of transverse sections around the thymus in adult SK2 medaka. (**A**) Control. (**B**) and (**C**) Dorsal irradiation by ion beam irradiation. (**D**) γ-Ray irradiation. (^E^ and ^F^) Ventral irradiation. Samples were fixed 3 days (**B** and **E**), and 7 days (**C**,**D**, and **F**) after irradiation. The dotted circles indicate the thymus. The box inside each panel shows an enlarged view of the cells in the thymus. The scale bars indicate 100 μm.

We started by checking several organs known to be sensitive to radiation to confirm the irradiation method and to analyse the effect of radiation on the whole body. The thymus, which is highly sensitive to radiation in both mammals and medaka, is situated in the medaka on both sides of the dorsal aspect of the branchial cavity^25^,^26^. The size of the thymus was reduced at 3 days after irradiation in the DR medaka (Fig. 2B) and did not recover until 7 days after irradiation (Fig. 2C). This reduction in tissue was similar to that observed in a γ-ray-irradiated thymus (Fig. 2D) reported in a previous study^26^. By contrast, the size of the thymus did not differ between the VR and control fish (Fig. 2A,E,F). The testis is also known to be a radiosensitive organ. Significant atrophy and sperm nuclei disturbance were observed in the DR and VR testis more severely than in the γ-ray-irradiated testis (Fig. S1), which confirmed a previous report that irradiation affects spermatogonial stem cells and spermatogenesis in the testis^18^. We also checked the phenotype in the skin and found acute indicators of local damage, such as hypertrophy and lifting of cells in the irradiated skin (Fig. S2).

The cells of the intestine are highly proliferative and are susceptible to radiation damage in mammals^27^. However, there were no obvious differences at the tissue or cellular level in the intestine among the DR-, VR-, and γ-ray-irradiated, and control fish at 3, 5, and 7 days after irradiation (Fig. S3).

### Reduction in blood cell mass in the irradiated kidney

**Figure 3:**
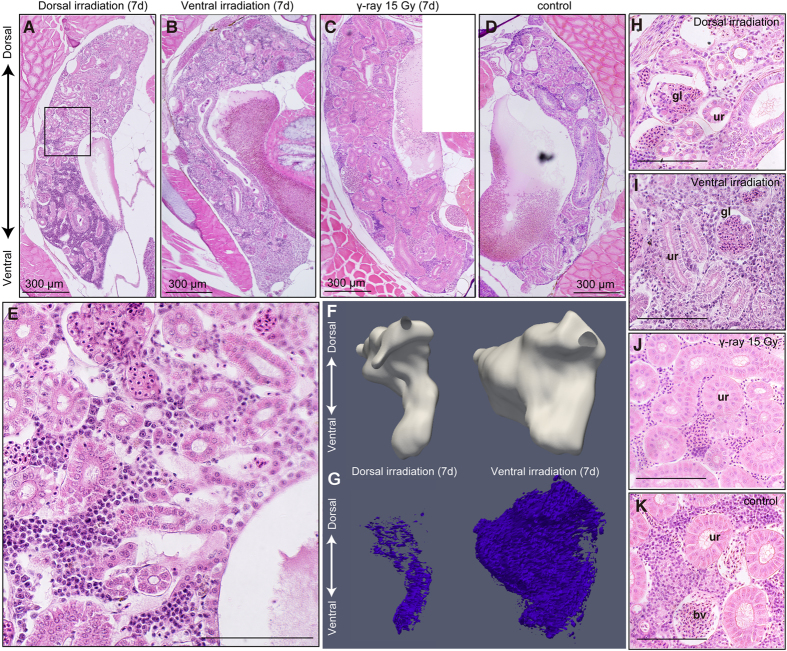
HE staining of transverse sections and 3D reconstruction around the kidney 7 days after dorsal or ventral irradiation with heavy-ion beam or γ-ray irradiation in adult medaka. (**A,E** and **H**) Dorsal irradiation with heavy-ion beam irradiation. An enlarged view of the square region is shown in (**B** and **I)** Ventral irradiation with heavy-ion beam irradiation. (**C** and **J**) γ-Ray whole-body irradiation. (**D** and **K**) Non-irradiated controls. (**F**) 3D reconstruction of a dorsally or ventrally irradiated kidney. (**G**) 3D reconstruction of blood cells in the same kidney as shown in (**F**). bv, blood vessel; gl, glomerulus; ur, uriniferous tubule. (**A**–**D**) The scale bars indicate 300 μm. (**E,H**–**K)** The scale bars indicate 100 μm.

In fish, the kidney works as a haematopoietic organ in addition to its osmoregulatory and urine-producing functions. The kidney of the medaka is located on the dorsolateral side at a depth of approximately 2 mm from the dorsal skin (Fig. 1D). In the DR fish, nucleated blood cells were absent from the dorsal region of the kidney, but this reduction in cell number was not observed in the kidney of VR fish (Fig. 3A,B). γ-Ray-irradiated medaka showed fewer blood cells in the whole kidney (Fig. 3C). To determine whether this blood cell reduction in the DR fish is due to the ion beam range, we also irradiated the lateral side of the fish. The irradiated kidneys showed a loss of blood cells, which suggested that carbon-ion irradiation directly damaged the haematopoietic stem cells and erythroid progenitors in the kidney (Fig. S4). Detailed observations showed the area 2 mm depth from the dorsal surface around the Bragg peak in the DR kidney (Fig. 3E and Fig. S5).

We performed three-dimensional (3D) reconstruction to analyse the volume and cell composition of the locally irradiated kidney. The dorsal part of the kidney, where the heavy-ion beam reached, showed severe atrophy in the DR fish compared with the VR fish at 7 days after irradiation (Fig. 3F). Blood cell in the DR kidney was significantly lower only in the irradiated area and was not reduced in the VR kidney (Fig. 3G). Because the kidney has a haematopoietic function in fish, it is likely that irradiation damaged the haematopoietic stem cells. These results suggest that the kidney shows a local response to irradiation.

Surprisingly, in both the DR and VR fish, the Bowman capsule in the kidney was oedematous (Fig. 3H,I). By contrast, there was no oedema in the Bowman capsule 7 days after γ-ray irradiation (Fig. 3J). The oedema observed seemed to be induced by irradiation with high-radiation biological effectiveness such as a carbon-ion beam. Oedema was observed in capillaries of the VR kidney even though these capillaries did not receive irradiation, which suggests that oedema was a secondary effect rather than a direct response to irradiation.

### Telangiectasia as a systemic radiation impact in the gill

**Figure 4:**
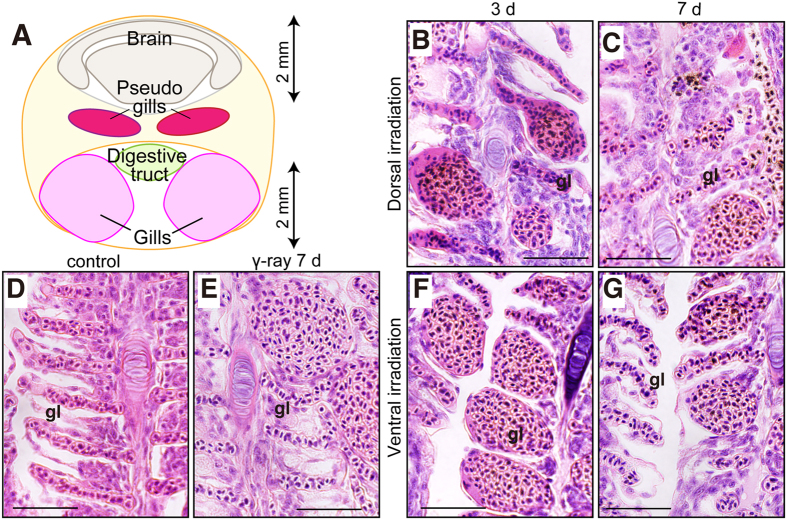
Phenotype in the branchial filament in the secondary gill lamella. (**A**) Diagram of the branchial position in the transverse section of medaka. (**B**–**G**) HE staining of transverse sections around the branchia in adult SK2 medaka. (**B** and **C**) Dorsal irradiation with heavy-ion beam irradiation. (**D**) Non-irradiated control. (**E**) γ-Ray whole-body irradiation. (**F** and **G**) Ventral irradiation. Samples were fixed 3 days (**B** and **F**) and 7 days (**C,E** and **G**) after irradiation. gl, secondary gill lamella. The scale bars indicate 50 μm.

The gills are located in the ventral region in medaka, have a large surface area and are surrounded by blood vessels, which provide gaseous exchange and excretion of nitrogenous waste products in fish (Fig. 4A). Obvious telangiectases or aneurysms of lamellae involved in haemorrhages were present throughout the pharyngeal arch. Haemorrhages were observed in the arterial capillaries of gill lamellae in both the VR and DR fish 7 days after irradiation (Fig. 4A–F). Blood accumulation in the arterial capillary of the pseudogills, which are located in the centre of the body, was also observed in both DR and VR fish (Fig. 4A and Fig. S6). The observation of capillary aneurysms in non-irradiated areas suggests that the oedema and haemorrhage are systemic effects.

### No effect of irradiation on heart rate

**Figure 5:**
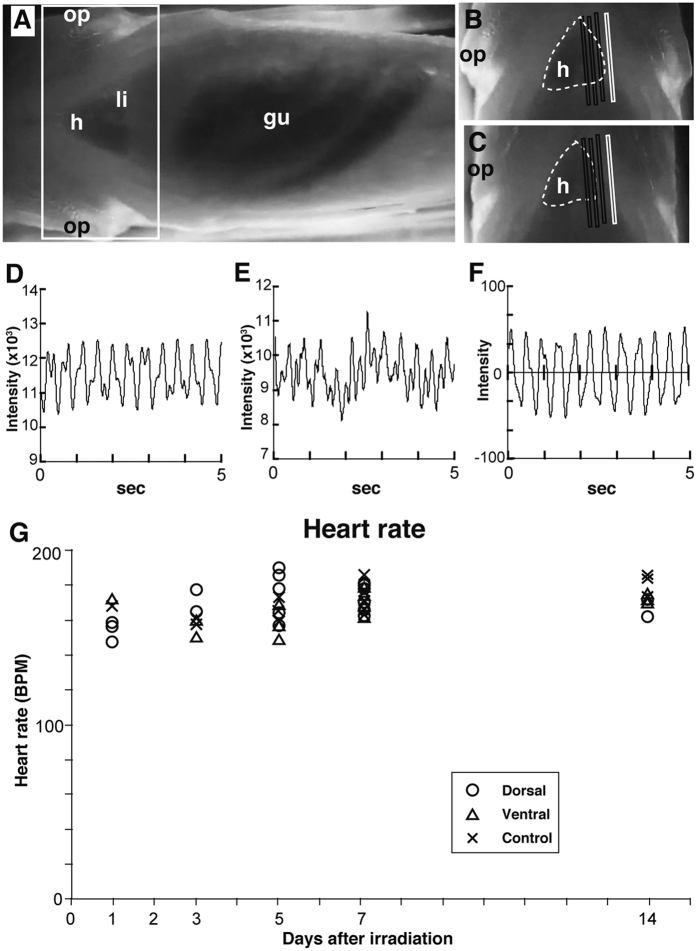
Steady-state heart rate after irradiation. (**A**) Example of a high-speed movie of the ventral view of an adult SK2 medaka. Images at diastole with opened opercula (**B**) and systole with closed opercula (**C**) in the heart area enclosed in the open white square in (**A**). The heart is traced in dashed white lines. Selected areas (black and white boxes) were used to calculate the pixel intensity of the ROI in the heart and body region, respectively. Example data from pixel intensity from the heart and body areas were arrayed and smoothed (a 5 s example from a 3 min sample; (**D** and **E)** respectively). Subtracted values for the difference between the time points in (**D**) from those in (**E**) show the heart movement intensities, which were processed further using a smoothing algorithm for a moving average from 21 frames to correct for peak detection errors and to represent the cardiac beat shown in (**F**). gu, gut; h, heart; li, liver; op, opercula. (**G**) Group-averaged steady-state heart rates are plotted under each condition. Circles, triangles, and crosses indicate dorsal irradiation, ventral irradiation, and control, respectively.

To consider whether the accumulation of erythrocytes in the capillaries was caused by abnormalities in the heart such as tachycardia, we analysed the heart rate by taking high-speed movies of the heart. Medaka strain SK2 lacks chromatophores, which allows the visualization of internal organs, including the heart, from the outside^28^. We videotaped the ventral aspect of medaka at 300 frames per second and used the images to analyse the heart rate under each of the irradiation conditions. The averages of the 3 min heart rates at 5 days after irradiation were 165 ± 5.35, 174 ± 12.9, and 169 ± 8.14 beats per minute (bpm) in the control (n = 3), DR (n = 5), and VR (n = 4) fish, respectively (p = 0.24). The average heart rates at 7 days after irradiation were 171 ± 7.78, 172 ± 6.99, and 170 ± 6.47 bpm in the control (n = 6), DR (n = 6), and VR fish (n = 4), respectively (p = 0.90; Fig. 5G). The heart rate did not differ significantly between the irradiated fish and controls. There were no morphological alterations in the myocardium in either the DR or VR fish. The lack of differences in heart rate and morphology suggests that there were no significant effects of irradiation on the heart in medaka (Fig. S3D–F and J–L).

## Discussion

We established a system with which to study heavy-ion beam irradiation in medaka. Using 2D and 3D analysis of serial sections of the whole medaka body, we observed both local responses and systemic responses to the irradiation. We also observed bleeding or oedema in capillaries outside the irradiated area without alterations in the heart rate, which suggests a systemic response caused by local irradiation.

Using the irradiation experiment facility of the TIARA at National Institutes for Quantum and Radiological Science and Technology in Takasaki, we established an experimental system for the localized carbon ion-beam irradiation of the dorsal or ventral aspects of individual medaka fish. Because of its small size compared to mice, medaka is a good model for observing the effects of irradiation in serial sections of the entire body^29^. Histological analyses of serial sections of the whole body enabled us to observe tissues that are difficult to isolate, such as blood vessels, and 3D reconstruction allowed us to examine localized irradiation effects in tissues. Although the phenotypes were evaluated in only a few target organs in this study, we have made all of the digital images available as an open source ( https://ds.cc.yamaguchi-u.ac.jp/~vs_08_2p/index.html) to others wishing to examine the other tissues.

The area between irradiated and non-irradiated in the actual sections were not distinct, as the calculated width of the Bragg peak was approximately 10 μm in the kidney. To obtain an explanation for this expanded area, transgenic medaka, which can detect DNA repair, will be useful. Because it is transparent, transgenic medaka provides a good system for analysing the effect *in vivo*. In addition, the TIARA has a microbeam irradiation system for biological targets, which can irradiate more limited parts, such as specific tissues, of the target organisms using a carbon-ion beam of the same ion energy^30^. Applying microbeam irradiation to the specific tissues of the medaka might be useful in studying the localized and systemic effects of irradiation in targeted tissues in detail.

The kidney in fish is a haematopoietic organ and contains erythroblasts and haematopoietic stem cells^31^. In the present study, atrophy and reduced blood cell numbers were observed in the dorsal part of the kidney in the DR fish and in the whole kidney in γ-ray-irradiated fish. These changes were not observed in the VR fish. These results suggest that irradiation had a systemic effect on the systematic circulation rather than a direct effect on the kidney.

A previous report showed that the cells in the thymus recovered 1 week after irradiation with 2 kR of γ-rays in medaka, but there was no recovery in the thymus irradiated with 4 kR of γ-rays^26^. Our study confirmed this finding. Taken together, these findings suggest that the thymus is highly sensitive to direct radiation exposure. Irradiation with a heavy-ion beam induced sloughing of epithelial cells on the irradiated side 3–5 days after the irradiation in the DR and VR medaka; the side that was not irradiated showed no such radiation defect.

In this study, the intestinal tract showed no effects of irradiation at the tissue level. The height of the folds of the intestine decreases after X-ray irradiation in medaka^32^,^33^. However, it may be difficult to detect these morphological alterations with HE staining in whole-body sections without dissection. Immunohistochemical analysis using cleaved-caspase 3 antibody will detect cellular alterations such as cell death.

The kidney is a haematopoietic organ in fish^31^. The phenotype in the capillaries did not differ between the DR fish with irradiated kidneys and VR fish, whose kidneys were not irradiated. This finding suggests that the damage observed in the capillaries represents secondary damage caused by irradiation of peripheral blood, including cytokine releases, and was not caused by the effects on haematopoietic stem cells in the kidney. In the kidney with oedema, the concentration of plasma increases via the retention of sodium. Consequently, the kidney venous pressure may increase. In this study, abnormality of the blood pressure of the capillary spread to non-irradiated areas caused by the kidney being damaged by irradiation.

## Methods

### Fish maintenance

Specimens of the medaka (*Oryzias latipes*) strain SukeSuke (SK2, *b*^*g8*^; null melanophore, *lf*; leucophore-free, *gu*; guanineless) reared in standard tanks were obtained from our breeding colony^28^. All experiments were conducted on adult fish, the total lengths of which were 2.7 ± 0.2 cm, more than 3 months after hatching. The fish were maintained under standard laboratory conditions at 26 °C with a 14:10-h light–dark cycle and fed a powdered diet (Tetra-min, Tetra Werke Co., Melle, Germany) and brine shrimp (*Artemia franciscana*) twice per day.

### Radiation dose calculation

The theoretical energy loss of ion beams and LET values at the target cells were calculated using the Energy Loss Modify (ELOSSM) code, a part of the Induced Radioactivity Analysis Code System (IRACM)^34^. For the broadbeam irradiation, a 60 mm × 60 mm area was irradiated uniformly by the raster-scanned beam, and the irradiation dose was calculated from the real-time simultaneous measurement value of electric current. Details of the broadbeam of carbon-ion exposure to biological samples were previously described^35^, and the calculated range of the carbon particles was approximately 2.2 mm in water (Fig. 1A).

### Radiation exposure

Irradiation experiments with a vertical heavy-ion beam were performed in an accelerated beam with an Azimuthally Varying Field cyclotron of Takasaki Ion Accelerator for Advanced Radiation Application (TIARA; Takasaki, Japan), QST^36^ ( www.researchgate.net/publication/229050081_Use_of_Microbeam_at_JAEA_Takasaki). For the carbon-ion beam irradiation, adult fish were anaesthetized with 0.02% MS-222 and aligned dorsally or ventrally in a polystyrene foam mould in a 60 mm petri dish with anaesthetic (Fig. 1B). The mould had a 6 mm-deep and 6 mm-wide groove to fit the fish, and each end of the fish was gently blotted with paper towels soaked with anaesthetic to restrict the movement of the fish; they were then covered with 8 μm-thick Kapton polyimide film (Dupont-Toray Co. Ltd., Tokyo, Japan) on top (Fig. S7). The fish were irradiated with a ^12^C^6+^ ion beam (26.7 MeV/u) in the TIARA at room temperature. The uniformity of irradiated particles was confirmed using CR-39 (Poly Allyl Diglicol Carbonate) membranes (Fig. S7). We monitored the movement of the fish during carbon ion beam irradiation and used the fish that did not move during irradiation. The linear energy transfer (LET) of the ion at the body surface was 78.4 keV/μm. The irradiated dose was 15 Gy at a dose rate of 0.75–1.50 Gy/sec. The irradiated dose was calculated according to the following equation: Dose (Gy = J/kg) = number of irradiated particles (/cm^2^) × LET (keV/μm) × 10E7(μm × cm^2^/kg) × 1.602E-16 (J/keV). There was an increase in the dose according to the LET around the Bragg peak (Fig. 1C in red). The calculated LET around the Bragg peak was approximately 800 keV/μm. The control fish were anaesthetized and placed in the petri dish but without irradiation. After irradiation, the fish were placed in a tank to recover from the anaesthetic. Both the VR and DR medaka survived at least 4 weeks after irradiation.

For the γ-ray irradiation, fish were subjected to whole-body irradiation from ^137^Cs γ-rays at the University of Tokyo in Kashiwa (Gammacell 3000 Elan, MDS Nordion, Ottawa, Canada) at room temperature in a plastic container with water without anaesthesia at doses of 15 at a dose rate of 0.12 Gy per second considering the decay of the radiation source.

### Histological examination

The medaka were anaesthetised and fixed in Davidson’s fixative solution (22% of a 37% solution of formaldehyde, 33% ethanol, 11.5% glacial acetic acid, and 33.5% distilled H_2_O) at room temperature. The fixed samples were dehydrated with ethanol, embedded in paraffin (Parabett, Lot No. 160101 Muto Pure Chemicals Co., Ltd., Tokyo, Japan), and cross-sectioned into a complete series of serial sections (5 μm) through the whole body using a sliding microtome (Retoratome, REM-700 Yamato Koki Industrial Co., Ltd., Saitama, Japan). Sections were stained with haematoxylin and eosin (HE) for light microscopy observation. Images of the HE-stained serial sections were acquired using a digital camera (DP-70, Olympus, Tokyo, Japan) mounted on a microscope (BX50, Olympus, Tokyo, Japan) with a 40× objective lens. Digital images were acquired with an imaging system (VS-100, Olympus, Tokyo, Japan). The main components of the system were microscopy (VS-BX, Olympus, Tokyo, Japan), a CCD camera (PIKE F-505, Allied Vision Technologies, Germany), and image control software (AWS, Olympus, Tokyo, Japan). We used two objective lenses, 4× and 40× (UplanApo, Olympus, Tokyo, Japan), for an overview image and high-resolution image, respectively. The target area was computed and divided into a number of rectangular areas with an adjacent overlap of 5% and was imaged using a tiling method. The JPEG format was chosen for image compression. The user can observe the high-resolution histological image as a huge (Giga size) seamless tiling image with a web browser according to the display resolution via the Internet. All digital images are available online ( https://ds.cc.yamaguchi-u.ac.jp/~vs_08_2p/index.html).

### 3D image construction

Images of the HE-stained serial sections were acquired using a digital camera (DP-70, Olympus, Tokyo, Japan) mounted on a microscope (BX50, Olympus, Tokyo, Japan) with a 10× objective lens. Images from single sections were joined together and aligned using Photoshop software (Adobe Systems Inc., San Jose, CA, USA). The area of the kidney was chosen manually using Photoshop software. Serial segmented images were stacked in order as XYZ images using ImageJ software^37^ and aligned automatically using the LPX-Registration program in Pixel ImageJ Plugins ( http://lpixel.net/services/research/lpixel-imagej-plugins/). The aligned XYZ images were rendered volumetrically in real time using ParaVie^38^.

### Heart rate analysis

Adult fish were placed in a pierced clear tank and were positioned inside the water bath, supplied with sufficient water and oxygen, and maintained at 25 ± 1 °C throughout heart movement recording. Experiments were performed from 14:00 to 17:00 in consideration of the diurnal rhythm of the autonomic nervous system. Heart movement in adult medaka was recorded by taking videos of the ventral view of the fish through the transparent peritoneum using an inverted stereomicroscope equipped with a digital high-speed camera (Exilim EX-F1, CASIO Computer Co., Ltd., Tokyo, Japan) at 2.0× magnification^39^. Digital pictures at 300 frames per second with a resolution of 512 × 384 pixels were captured for up to 20 min and recorded on a PC using Final Cut Pro software (Apple Inc., Cupertino, CA, USA).

Three rectangular regions of interest (ROI) were selected in the heart region within the image of the fish abdomen, and one ROI was placed outside of the heart region to serve as a reference for body movement attributable to ventilation. The pixel intensities of each ROI were digitalized throughout the entire time series examined using Bohboh software (Bohboh Soft, Tokyo, Japan) and processed further using Cutwin mathematical software (EverGreen Soft, Tokyo, Japan). The pixel intensity of the ROI outside the heart region was subtracted from the mean intensity of the three ROIs in the heart image for each frame throughout the entire time series of images to remove the contribution of body movement caused by ventilation (Fig. 5A–C). Data were processed by taking a moving average over 21 frames. The period between pixel intensity minima, representing the end of diastole, provided the interbeat interval from which we calculated the beat-by-beat heart rate (Fig. 5F). We then averaged the beat-by-beat heart rates during collection for 3 min to generate steady-state heart rates^40^.

### Statistical analysis

The means, standard deviations, and ranges of the experimental data were determined. One-way analysis of variance was used to analyse the data using Microsoft Excel software (Microsoft Co., Redmond, WA, USA) at the 0.05 significance level. Tukey’s HSD test was used for multiple comparisons.

### Ethics statement

All experiments were performed in accordance with the Japanese laws and guidelines for the care of experimental animals according to the University of Tokyo Animal Experiment Enforcement Rule. The protocol was approved by the Committee on the Ethics of Animal Experiments of the University of Tokyo (Permit Number: C-14-02). All surgery was performed under MS-222, and all efforts were made to minimize suffering.

## Additional Information

**How to cite this article**: Nagata, K. *et al*. *In vivo* 3D analysis of systemic effects after local heavy-ion beam irradiation in an animal model. *Sci. Rep.* **6**, 28691; doi: 10.1038/srep28691 (2016).

## Supplementary Material

Supplementary Information
