# Supplementary material for: In vivo 3D analysis of systemic effects after local heavy-ion beam irradiation in an animal model
Source: Sci Rep. 2016 Jun 27;6:28691. doi: 10.1038/srep28691 (PMC4922018; doi:10.1038/srep28691)
Supplement: Supplementary Information [file srep28691-s1.doc]

**Title**: *In vivo* 3D analysis of systemic effects after local heavy-ion beam irradiation in an animal model.

# Authors: Kento Nagata^1#^, Chika Hashimoto^1#^, Tomomi Watanabe-Asaka^1*^, Kazusa Itoh^1^, Takako Yasuda^1^, Kosaku Ohta^1^, Hisako Oonishi^1^, Kento Igarashi^1^, Michiyo Suzuki^2^, Tomoo Funayama^2^, Yasuhiko Kobayashi^2^, Toshiyuki Nishimaki^3^, Takafumi Katsumura^3^, Hiroki Oota^3^, Motoyuki Ogawa^3^, Atsunori Oga^4^, Kenzo Ikemoto^4^, Hiroshi Itoh^4^, Natsumaro Kutsuna^1, 5^, Shoji Oda^1^, and Hiroshi Mitani^1^

**Affiliations**: ^1^Department of Integrated Biosciences, Graduate School of Frontier Sciences, The University of Tokyo, Chiba, Japan; ^2^Takasaki Advanced Radiation Research Institute, Quantum Beam Science Research Directorate, National Institutes for Quantum and Radiological Science and Technology, Gunma, Japan; ^3^ Department of Anatomy, Kitasato University School of Medicine, Kanagawa, Japan; ^4^Department of Molecular Pathology, Yamaguchi University Graduate School of Medicine, Yamaguchi, Japan; ^5^LPixel Inc., Tokyo, Japan; ^#^Equally contributed

***Corresponding author**: Department of Integrated Biosciences, Graduate School of Frontier Sciences, The University of Tokyo, 5-1-5 Kashiwanoha, Kashiwa, Chiba, 277-8562 Japan

Tel: +81-4-7136-3663; FAX: +81-4-7136-3669; E-mail: twatana@ib.k.u-tokyo.ac.jp

**
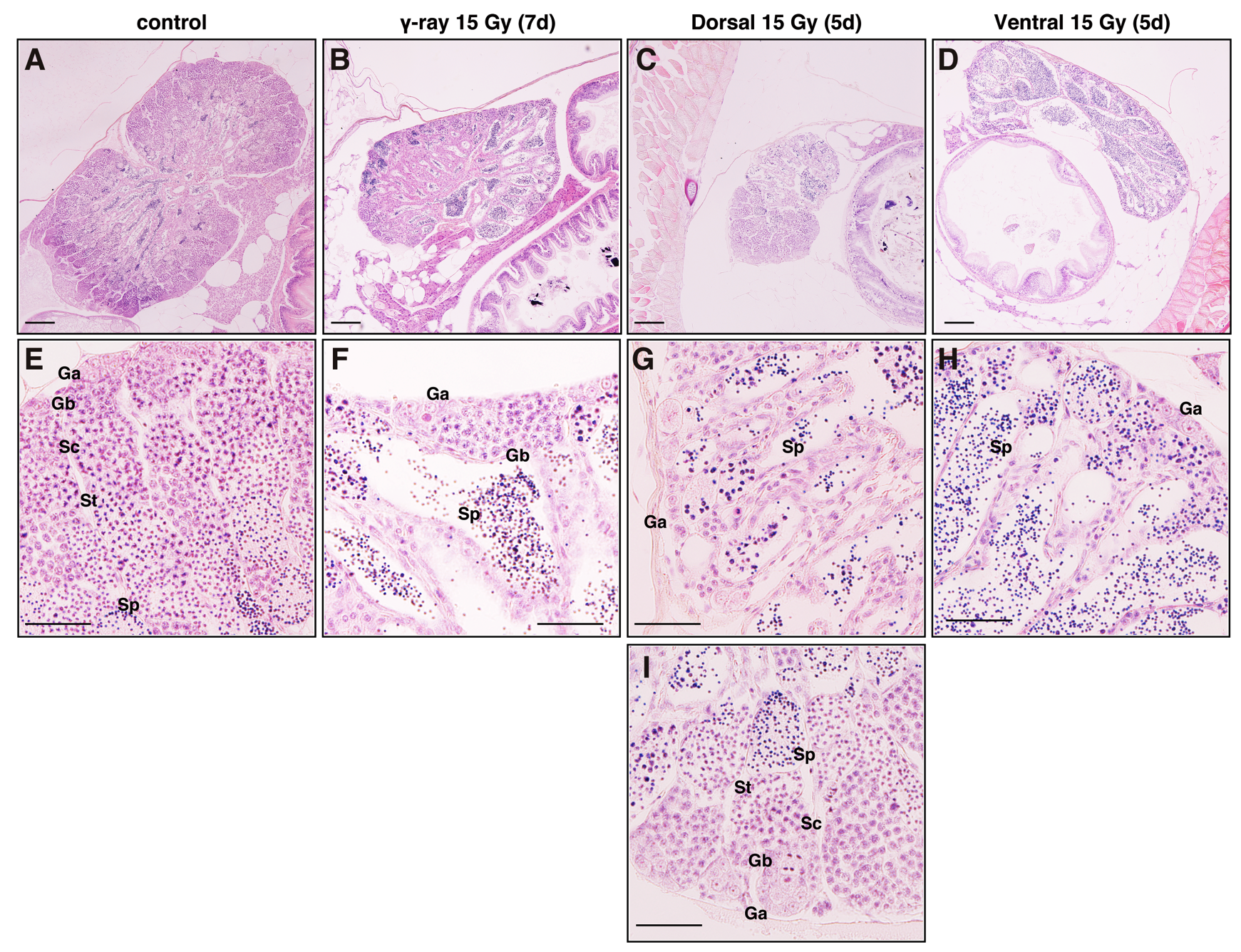
**

**Supplemental Fig. 1**

HE staining of transverse sections of the testis in adult SK2 medaka after dorsal or ventral irradiation with heavy-ion beam or γ-ray irradiation. A and E: Unirradiated control. B and F: Seven days after γ-ray irradiation. C, G and I: Dorsal irradiation with heavy-ion beam irradiation. The testis shows both irradiated (G) and non-irraiated (I) phenotype depend on the area. D and H: Ventral irradiation with heavy-ion beam irradiation. Ga: Cyst of A type spermatogonia, Gb: Cyst of B type spermatogonia, Sc: Spermatocytes, St: Spermatids, Sp: sperm. Scale bars indicate 50 μm.


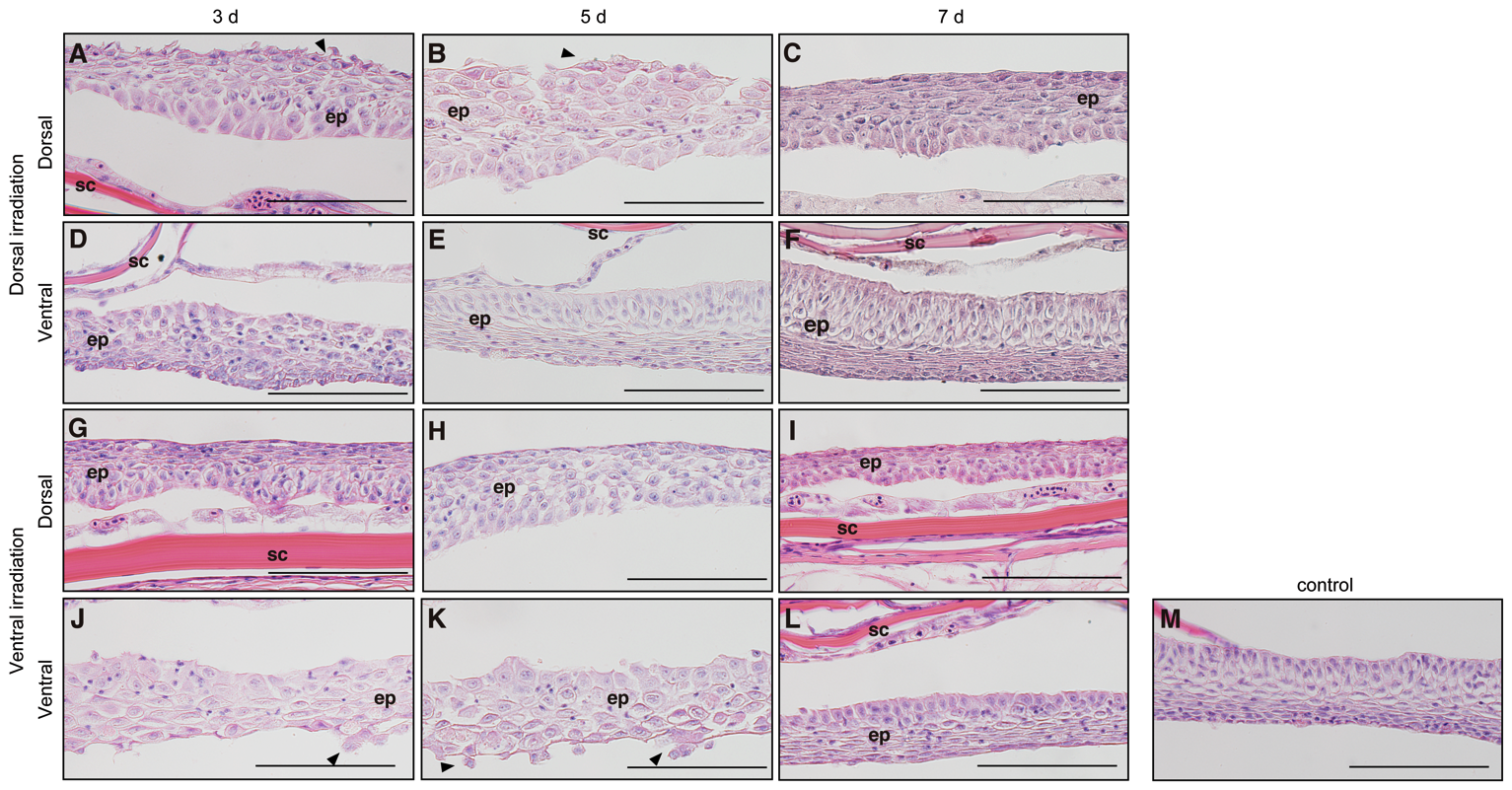
**Supplemental Fig. 2**

HE staining of transverse sections around the dorsal and ventral skin after dorsal or ventral irradiation with heavy-ion beam irradiation in adult SK2 medaka. A–C and G–I: Dorsal skin. D–F and J–L: Ventral skin. A–F: Dorsal irradiation with heavy-ion beam irradiation. G–L: Ventral irradiation with heavy-ion beam irradiation. Samples were fixed at 3 days (A, D, G, and J), 5 days (B, E, H, and K), and 7 days (C, F, I, and L) after irradiation. M: control. Arrowheads indicate the sloughing of cells. ep, epidermis; sc, scales. Bars indicate 50 μm.

**Supplemental Fig. 3**


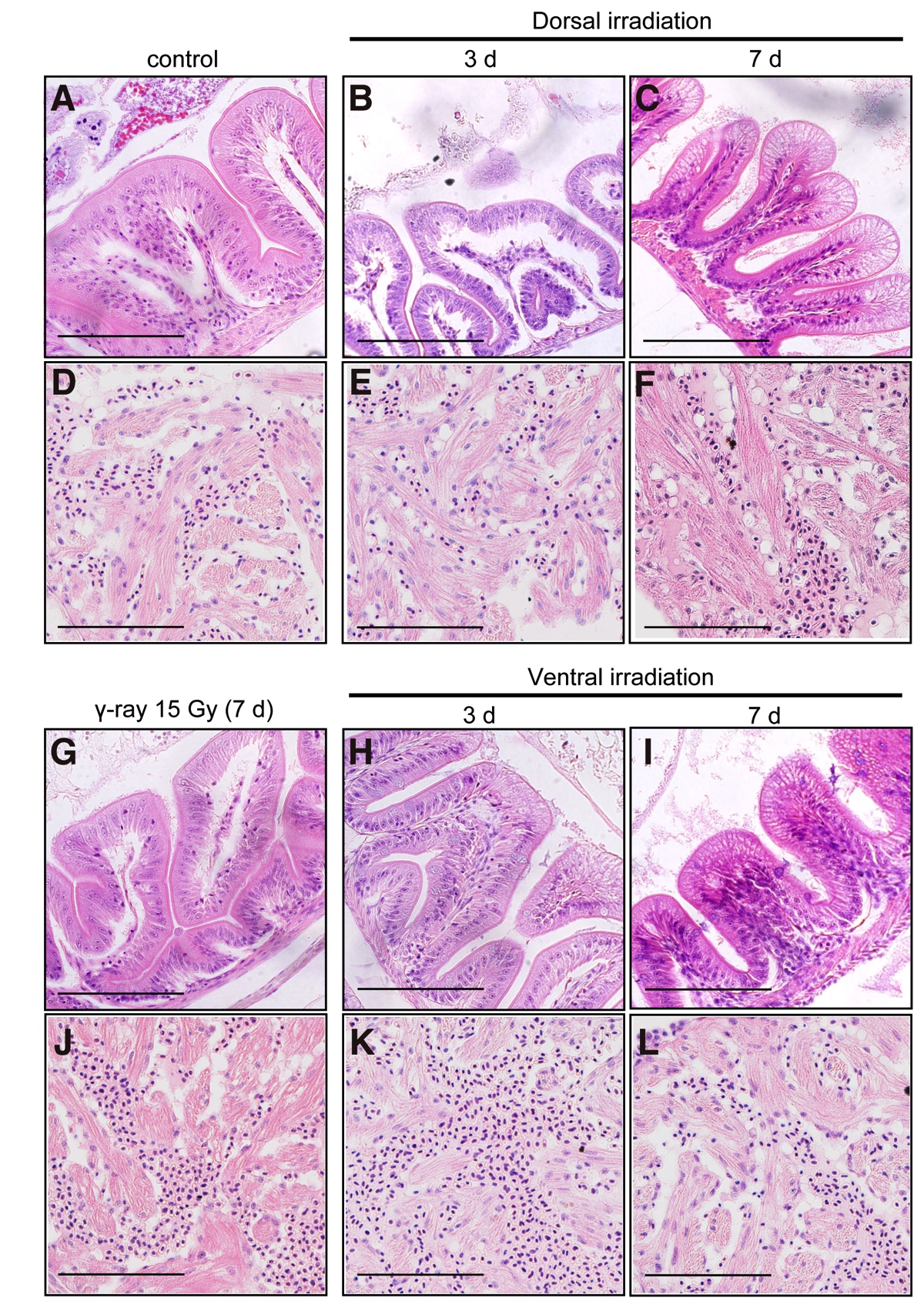
HE staining of transverse sections of the intestine and the heart in adult SK2 medaka. A–C and G–I: Intestine. D–F and J–L: Heart. A and D: Control. B, C, E, and F: Dorsal irradiation with ion-beam irradiation. G and J: γ-Ray irradiation. H, I, K, and L: Ventral irradiation. Samples were fixed 3 days (B, E, H, and K) and 7 days (C, F, G, I, J, and L) after irradiation. Scale bars indicate 100 μm.


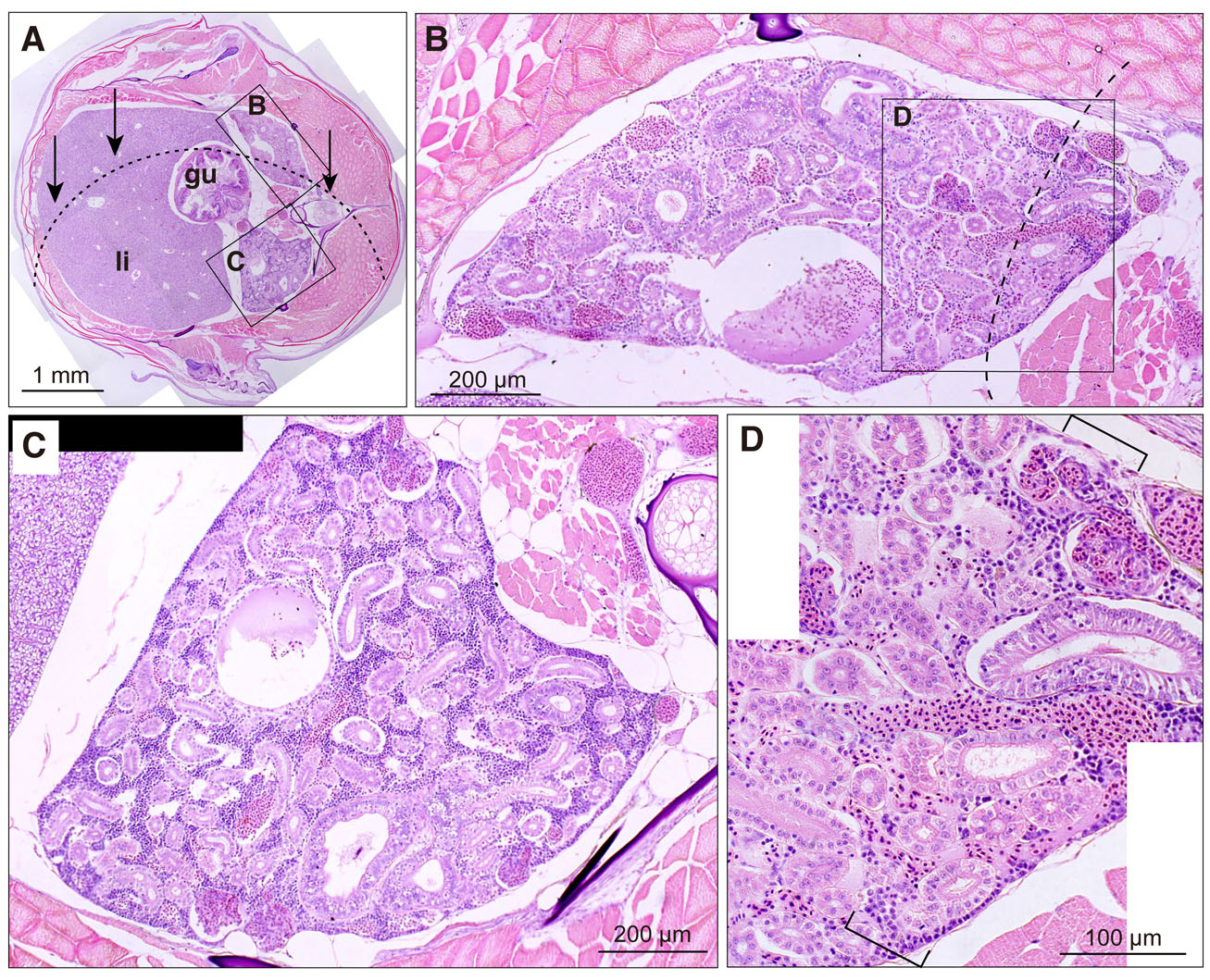
**Supplemental Fig. 4**

HE staining of transverse sections around the kidney in adult SK2 medaka 7 days after lateral irradiation with heavy-ion beam irradiation. A: Typical transverse section of the laterally irradiated medaka. Enlarged views of boxes B and C are shown in panels B and C. B: Partially irradiated side of the kidney. Enlarged view of the box is shown in panel D. C: Unirradiated side of the kidney. D: Enlarged view around the expected beam range. gu, gut; li, liver; Scale bars indicate 1 mm in A, 200 μm in B and C, and 100 μm in D. Dashed line indicates the expected range of the carbon-ion beam. Arrows show the irradiated side. Brackets indicate the expected beam range.


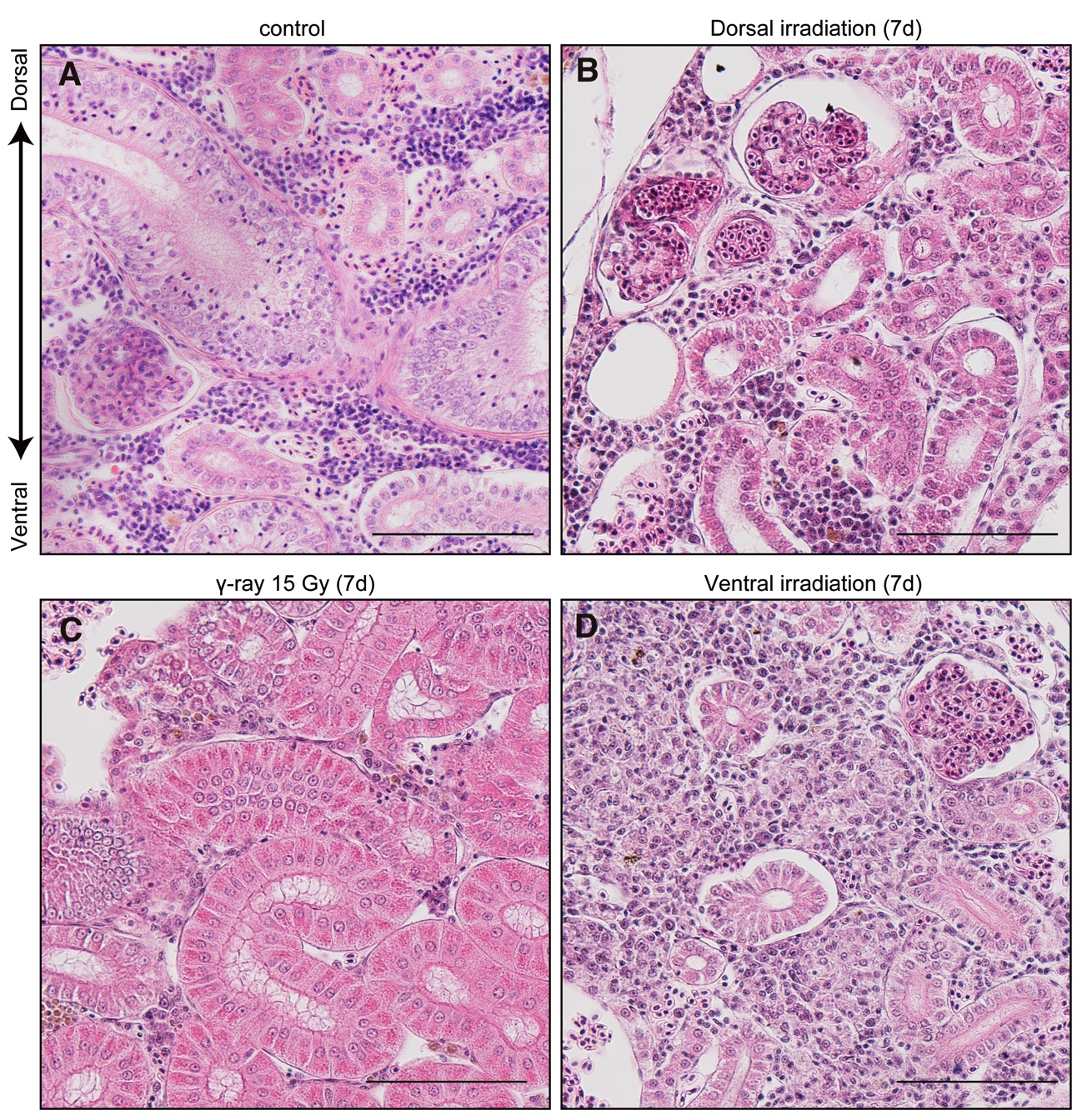
**Supplemental Fig. 5**

HE staining of transverse sections in the kidney 7 days after dorsal or ventral irradiation with heavy-ion beam or γ-ray irradiation in adult SK2 medaka. A: Unirradiated control. B: Dorsal irradiation. C: γ-Ray-irradiated fish. D: Ventral irradiation. Scale bars indicate 100 μm.


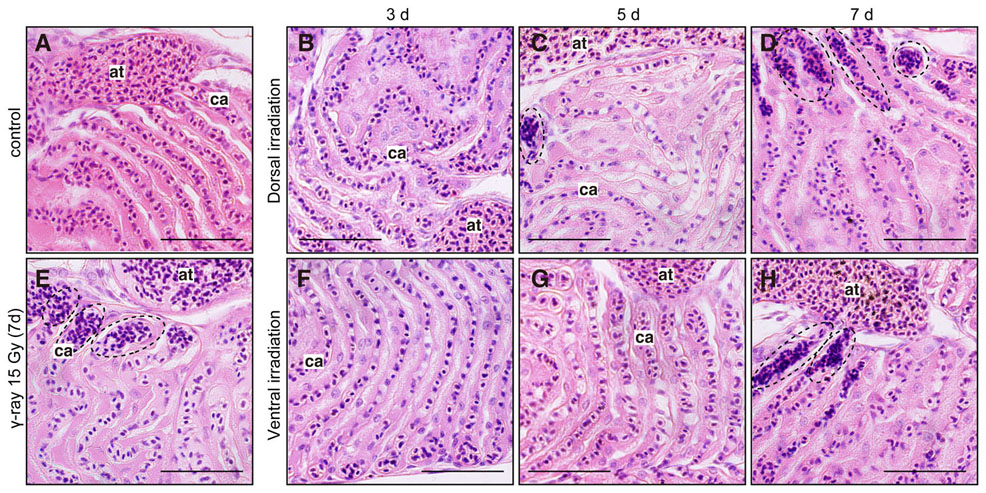
**Supplemental Fig. 6**

HE staining of transverse sections around the pseudobranchia in adult SK2 medaka. A: Unirradiated control. B–D: Dorsal irradiation. E: γ-Ray-irradiated fish. F–H: Ventral irradiation. Samples were fixed 3 days (B and F), 5 days (C and G), and 7 days (D, E, and H) after irradiation. at, artery; ca, capillary. Dashed circles indicate excess pooled blood in the capillary. Scale bars indicate 50 μm.

**
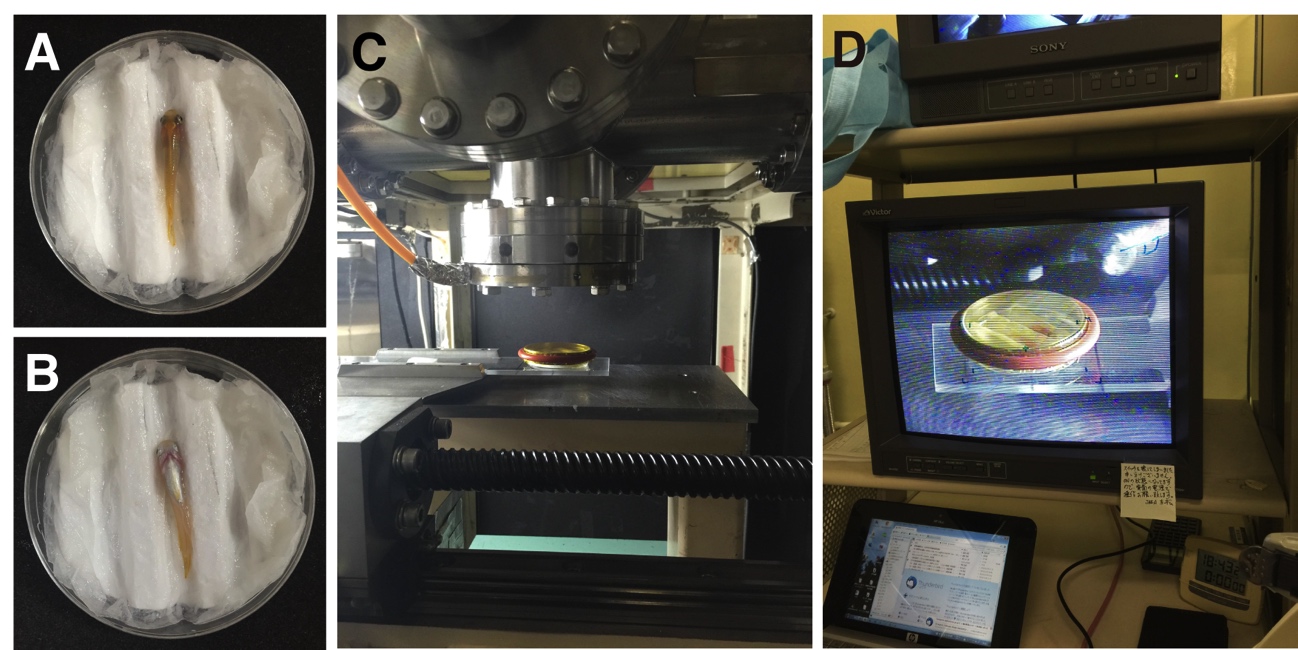
**

**Supplemental Fig. 7**

Experimental procedure for local irradiation. Medaka was anaesthetised and aligned as dorsally (A) or ventrally (B) on top in a mold. Irradiation was done by remote manipulation and the operator can observe the status of the sample during the irradiation (C and D).
